# National guidelines for diagnosis and treatment of osteoporosis in Slovakia

**DOI:** 10.1007/s11657-025-01538-z

**Published:** 2025-05-04

**Authors:** Juraj Payer, Peter Jackuliak, Peter Vaňuga, Zdenko Killinger, Soňa Dubecká, Martin Kužma

**Affiliations:** 1 5th Department of Internal Medicine, Faculty of Medicine, Comenius University, University Hospital Bratislava, Ružinovská 6, 82101 Bratislava, Slovakia; 2 Slovak Society for Osteoporosis and Metabolic Bone Disorders, Bratislava, Slovakia; 3 National Institute of Endocrinology and Diabetology, Lubochna, Slovakia

**Keywords:** Osteoporosis, Fracture, Prevention, Therapy, Vitamin D, Calcium, Antiresorptive therapy, Osteoanabolic therapy

## Abstract

**Background:**

Osteoporosis is a chronic, systemic skeletal disease characterized by decreased bone mass and microarchitectural deterioration, leading to increased fracture risk. In Slovakia, its prevalence is estimated at 6%, with substantial health, social, and economic burdens.

**Objective:**

The Slovak national guideline provides an overview for the diagnosis, prevention, and treatment of osteoporosis in Slovakia, reflecting recent scientific advances and recommendations from international bodies.

**Methods:**

The guidelines were developed by a multidisciplinary expert panel and officially adopted by the Ministry of Health of the Slovak Republic. They are based on current evidence and international standards, including FRAX, IOF, ISCD, and ESCEO recommendations.

**Results:**

Diagnosis involves clinical risk assessment, biochemical testing, and imaging—primarily DXA and trabecular bone score. FRAX with or without BMD enhances risk stratification. Osteoporosis is categorized as primary or secondary. Prevention strategies include lifestyle modification, calcium and vitamin D supplementation, and fall risk reduction. Pharmacologic treatment includes antiresorptive agents (bisphosphonates, denosumab, SERMs), osteoanabolic (teriparatide, romosozumab), and hormone therapy when indicated. Sequential treatment strategies are emphasized, particularly in high-risk individuals. Treatment monitoring includes bone turnover markers and periodic DXA.

**Conclusions:**

The Slovak guidelines provide a comprehensive and pragmatic approach for the management of osteoporosis across all stages, emphasizing early diagnosis, personalized treatment, and long-term fracture prevention. They align with European and global best practices and support clinical decision-making across specialties.

**Supplementary Information:**

The online version contains supplementary material available at 10.1007/s11657-025-01538-z.

## Definition and epidemiology

Osteoporosis is a systemic disease of the skeleton, defined as a decrease in bone mass content and disruption of the microarchitecture of bone tissue, causing increased bone fragility. The consequence is an increased risk of fractures even with minimal trauma [1]. The prevalence of osteoporosis in the Slovak population in accordance with SCOPE21 is estimated at ca. 6% [2]. The most common sites of typical osteoporotic fractures are the distal forearm, vertebrae, and the hip. The risk of osteoporotic fracture at these sites in postmenopausal women is ca. 40 to 50%, and in older men, the risk can range from 13 to 25%. As life expectancy increases, the incidence of osteoporotic fracture may increase. These fractures occur in the absence of any apparent trauma. Mortality in patients with vertebral and proximal femur fractures is ca. 6 to 8 times higher than in the healthy population, up to 20% of proximal femur fracture patients die within 1 year of the fracture, and another 30% remain permanently immobile. Osteoporotic fractures substantial affect morbidity, mortality, and quality of life [2].

### Classification

Osteoporosis can be divided into primary and secondary.

1. Primary osteoporosis
  Postmenopausal osteoporosis (type I)
  Senile osteoporosis (type II)
  Juvenile osteoporosis
  Idiopathic osteoporosis
2. Secondary osteoporosis is mostly caused by a known disease. It includes an entire range of disease and morbid conditions that lead to changes in bone metabolism.

#### Postmenopausal osteoporosis

Its appearance is characteristic ca. 15–20 years after menopause, and trabecular bone is most affected. Estrogen deficiency is the decisive pathophysiological mechanism of postmenopausal osteoporosis. The main clinical manifestation is bone fractures with a higher proportion of trabecular bone, mainly vertebrae.

#### Senile osteoporosis

It occurs mostly in people over 70 years of age. It affects women only slightly more often than men. The most significant pathogenetic factors of senile osteoporosis include age-decreasing bone formation, circulatory and neurotrophic bone disorders, age-related structural changes in bone collagen, and secondary hyperparathyroidism with reduced calcium absorption due to reduced calcitriol formation. It is manifested by fractures, both in ​​cortical and trabecular bone, mainly fractures of the proximal femur.

#### Juvenile osteoporosis

It affects children in prepubertal and adolescent age without any other underlying disease.

#### Idiopathic osteoporosis 

It occurs in younger individuals (both men and women) without a proven connection to another underlying disease.

The most common causes of secondary osteoporosis include the following:

- Osteoporosis with hormone deficiency: sex hormone and growth hormone deficiency
- Osteoporosis due to an excess of hormones: hypercortisolism, hyperthyroidism, hyperprolactinemia, hyperparathyroidism, and acromegaly
- Osteoporosis caused by nutritional disorders
- Insufficient intake of calcium, vitamin D, digestive disorders, and malabsorption syndromes
- Mineral and bone disorder in chronic kidney disease–mineral and bone disorder (CKD–MBD)
- Osteoporosis caused by inactivity
- Osteoporosis in chronic inflammatory diseases
- Osteoporosis in cancer
- Osteoporosis induced by medication

### Clinical manifestation

A fracture is commonly the first manifestation of osteoporosis. Typical osteoporotic fractures are fractures of the proximal femur, wrist, and vertebral fractures. Vertebral fractures can occur acutely or gradually. Acutely formed fractures are manifested by sudden sharp pain; otherwise, vertebral fractures present with chronic static back pain and loss of height. Up to 2/3 of vertebral fractures may be asymptomatic.

Other *osteoporotic fractures* are low-traumatic fractures of the humerus, pelvis, ribs, and—especially in patients with obesity and type 2 diabetes mellitus (DM)—also fractures of the distal part of the upper and lower limbs. Skull fractures and fractures of small bones of the hands and feet are not usually considered osteoporotic fractures.

The *uncontrollable risk factors* include age, female sex, genetic predisposition, white race, slender habitus, positive family history of osteoporotic fractures, and a history of a fracture after inadequate trauma. *Controllable risk factors* include lifestyle factors, chronic diseases, and medications with a negative effect on bone metabolism (Table 1) [3].

**Table 1:** Conditions, diseases, and medications causing or leading to osteoporosis and fractures

Lifestyle factors	• Low intake of calcium in the diet • Insufficient vitamin D intake • Lack of physical activity or immobility • Smoking • Alcohol consumption (≥ 3 units per day) • Tendency to falls • Malnutrition or low body weight (BMI ≤ 19 kg/m^2^)
Endocrine disorders	• Premature ovarian failure • Hypogonadal conditions • Panhypopituitarism • Anorexia nervosa and bulimia • Turner and Klinefelter syndrome • Hyperprolactinemia • Adrenal insufficiency • Hyperparathyroidism • Hypercorticism • Thyrotoxicosis • Type 1 and type 2 DM • Acromegaly
Gastrointestinal disorders	• Celiac disease • Malabsorption • Non-specific inflammatory bowel diseases • Condition induced by bariatric surgery • History of gastrointestinal bypass operations • Severe impairment of liver and pancreas function
Hematologic and rheumatologic disorders	• Hemophilia • Sickle cell anemia • Leukemia and lymphomas • Systemic mastocytosis • Multiple myeloma • Thalassemia • Autoimmune inflammatory rheumatic diseases
Other disorders	• Epilepsy • Idiopathic scoliosis • Amyloidosis • Multiple sclerosis • Chronic metabolic acidosis • Muscular dystrophy • Congestive heart failure • Parenteral nutrition • Depression • Post-transplant conditions • Chronic obstructive pulmonary disease • Previous fracture in adulthood • Kidney diseases with limitation of their function • Sarcoidosis
Medication	• Glucocorticoids (> 5 mg/day of prednisone, longer than 3 months, or a cumulative dose of 2.7 g of prednisone per year) • Androgen-deprivation therapy • Aromatase inhibitors; anti-estrogenic treatment • Gonadotropin-releasing hormone agonists • Anticoagulants • Proton pump inhibitors • Glitazones • Lithium • Cyclosporin A and tacrolimus • Barbiturates • Anticonvulsants • Chemotherapeutics in antitumor treatment • Depot medroxyprogesterone

### Diagnostics: procedure for determining the diagnosis

Diagnostics and differential diagnosis are provided by a bone-focused physician specializing in the fields of rheumatology, endocrinology, orthopedics, internal medicine, gynecology, or clinical oncology. Other physicians: specialists carry out consulting activities for the needs of diagnosis, differential diagnosis, and treatment, primarily for secondary osteoporosis and other metabolic diseases of the skeleton.

Diagnosis and differential diagnosis of osteoporosis consists of the following:

- History-taking
- Physical examination
- Laboratory tests
- Imaging examinations
- Determination of fracture risk using the FRAX calculator

## History

The aim of the history is to obtain information about the fractures overcome so far and the circumstances of their occurrence, data on fractures in family members, dietary habits with a focus on ensuring the supply of calcium and vitamin D, and data on physical activity. Considering the asymptomatic course of the disease, it is necessary to focus on the determination of osteoporosis risk factors and fracture risks (see previous section) [3] and the symptoms of diseases leading to secondary osteoporosis.

## Physical examination

In the physical examination, one should focus on the change in height (decrease in height by more than 4 cm compared with the height in youth), accentuation of thoracic kyphosis, bulging of the abdomen, low body weight (BMI: below 19 kg/m^2^), gracile habitus, physical findings indicating for another disease that can lead to secondary osteoporosis, and a physical finding typical of another metabolic osteopathy.

## Laboratory tests

The aim of the biochemical examination is as follows:

- Assessment of the state of phospho-calcium metabolism.
- Evaluation of the activity and speed of bone metabolism.
- Differential diagnosis of secondary osteoporosis and other skeletal diseases.

Basic laboratory tests in the diagnosis and differential diagnosis of osteoporosis are as follows:

- Blood count
- C-Reactive protein (CRP)
- Serum calcium, phosphorus, and alkaline phosphatase
- Albumin
- Aspartate aminotransferase (AST), alanine aminotransferase (ALT), and gamma-glutamyl transferase (GMT)
- Glomerular filtration (GF) and creatinine
- Protein electrophoresis (ELFO) in serum and urine
- Excretion of calcium and phosphates in the urine in 24 h (or their fractional excretions)
- Examination of the level of 25-hydroxy vitamin D3
- Examination of bone turnover markers

The reference marker of bone formation is the serum level of P1 NP (procollagen type I N propeptide), and the reference marker of bone resorption is the CTX (C-terminal cross-linking telopeptide of type I collagen). Examination of markers of bone turnover is important:

- Prediction of the rate of bone mineral density (BMD) decline.
- Fracture risk prediction independent of BMD.
- Evaluation of the correct use of anti-osteoporotic treatment (compliance).
- Assessment of the duration of the interruption of therapy (so-called drug holiday).
- Measurement of bone turnover markers is indicated:
- Before starting anti-osteoporotic therapy.
- After 3 to 6 months from starting the treatment.
- During a planned interruption (drug holiday) or termination of treatment.

## Imaging examinations

The gold standard for measuring bone mass content is *densitometry* [4]. The principle of densitometric examination consists in measuring the degree of absorption of X-ray rays by bone tissue (RTG absorptiometry: DXA, from English dual-energy X-ray absorptiometry). Indications for the densitometric examination are in Suppl. Table 3. The so-called *central densitometry* (DXA) measures BMD in the proximal femur, lumbar spine, and forearm.

In postmenopausal women and men over 50 years of age, evidence of a decrease in bone density T-score ≤ − 2.5 SD is required in at least one of the following locations: antero-posterior (AP) projection of lumbar spine or proximal femur. A T-score is a multiple of a standard deviation from the mean of a normal young healthy population. The lowest values ​​of the measured T-score are always considered. When measuring in ​​the lumbar spine, vertebrae L1 to L4 are evaluated. Vertebrae distorted by local structural changes must be excluded from the average, but at least two vertebrae must be analyzed. It is not possible to diagnose osteoporosis from the density of one vertebra. See also Suppl. Table 4 .

In premenopausal women and men younger than 50, it is appropriate to use the Z-score for the diagnosis of osteoporosis. The Z-score is a multiple of the standard deviation from the mean of a normal healthy population of the same age and sex. Osteoporosis can be diagnosed if low BMD (Z-score ≤ − 2.0 SD) is also associated with secondary causes of osteoporosis or if an osteoporotic fracture is already present (a vertebral fracture or two peripheral fractures after inadequate trauma). The diagnosis of osteoporosis cannot be established based on DXA measurements alone, and a Z-score of ≤ − 2.0 SD alone should be evaluated only as a decrease in BMD below the expected value for the given age and not as osteopenia or osteoporosis. A Z-score above − 2.0 SD should be evaluated as a BMD value within the expected range.

Measurements on the forearm can be used to diagnose osteoporosis in patients with the following:

- Primary hyperparathyroidism
- Extreme obesity (exceeding the limits of the densitometric table)
- No measurable or evaluable region of the spine or femur

To establish the diagnosis of osteoporosis, in the case of measurement on the forearm, it is necessary to evaluate the density in the area referred to as 33% radius (1/3 radius) of the non-dominant upper limb [5].

Ultrasound measurement of bone density in the heel region has only predictive value for fracture risk assessment. The measurement is not suitable for establishing a diagnosis of osteoporosis. Measurement of bone density using quantitative computed tomography (QCT) densitometry can be indicated if it is not possible to perform or evaluate the measurement by central DXA, and the patient’s history and clinical manifestations strongly suspect osteoporosis with the perspective of the need for anti-osteoporotic treatment. If the probability of fracture assessed by QCT of the spine (using device-specific thresholds) together with adverse clinical risk factors is sufficiently high, the indication of pharmacological treatment can be considered as measured by the DXA method [6].

A follow-up DXA study should be performed on the same device. If the device changes during the control measurement, it is not possible to evaluate the change in density without using it adequate conversion of the so-called cross-calibration. To assess the change in bone density, DXA measurement in the AP projection in ​​the lumbar spine or the area of ​​the proximal femur is recommended. The doctor should indicate the control measurement of the density at a time when it can be assumed that the change in bone density will be higher than the value of the least significant change (LSC) determined at the given workplace. Control measurement is indicated after 1 year of starting or changing anti-osteoporosis treatment. After proving the sufficient effectiveness of the applied treatment (see also the chapter on the evaluation of the effectiveness of the treatment), it is usually possible to extend the measurement interval, provided that the applied treatment is continued and the (presumed) adequate adherence of the patient to the treatment is ensured, and at the same time, the risk profile does not significantly change. If there is a reasonable assumption that a significant change in bone density will be achieved earlier than 1 year (rapid loss of bone density, e.g., in secondary osteoporosis, especially glucocorticoid-induced osteoporosis), it is possible to shorten the control measurement interval to less than 1 year. After completion or interruption of treatment, a control measurement is indicated after 18 to 36 months. Central densitometry in the forearm area—or ultrasound densitometry in the heel area—is not suitable for monitoring changes in bone density.

The evaluation of the densitometric measurement should be performed by a medical specialist with experience in the evaluation of densitometric scans based on the previous criteria. Osteoporosis is a lifelong disease with a persistent risk of fractures. Therefore, even if the T-score value improves and reaches the osteopenia or normal range during the treatment of osteoporosis, the diagnosis of osteoporosis remains [7].

### Requirements for the quality of densitometric workplaces

The quality control (QC) program should be in accordance with the manufacturer’s recommendation for system maintenance. If it is not recommended by the manufacturer, the following QC procedures are suitable: periodic (at least once per week) scanning of the phantom as an independent assessment of system calibration, printing, and analysis of data on calibration and phantom scans and verification of the average BMD of the phantom after each service intervention in the densitometer. Assessment of measurement accuracy is part of standard clinical practice. Each densitometric workplace should have its own calculated value of the minimum significant change in density, the so-called LSC, for each operator and after each device change. The measurement error specified by the manufacturer should not be used. In accordance with the ISCD, the acceptable value of the minimum significant change (i.e., the LSC) for individual sections and each operator should not be higher than 5.3% on the spine in the AP projection, higher than 5.0% in the area referred to as “total hip,” and higher than 6.9% on the neck of the femur. See also Suppl. Tables5 and 6 for** s**pecific requirements for the densitometric evaluation reports.

### Trabecular bone score

Trabecular bone score (TBS) is a tool that captures information regarding the trabecular microarchitecture and thus the quality of the bone structure based on the texture of the lumbar spine scans performed using DXA. Low TBS is significantly associated with the incidence of osteoporotic fractures and is partially independent of clinical risk factors and BMD in the area of ​​the lumbar spine and proximal femur. TBS may also play a role in assessing fracture risk in some causes of secondary osteoporosis in which bone density is poorly correlated with this risk (e.g., type 2 DM, hyperparathyroidism, acromegaly, and glucocorticoid-induced osteoporosis). Using TBS to determine fracture risk based on the FRAX calculator leads to improvement of fracture prediction.

TBS values are divided as follows: TBS ≥ 1.31 is considered normal; TBS from 1.23–1.31 is considered consistent with a partially degraded microarchitecture, and TBS ≤ 1.23 is defined a degraded microarchitecture [8].

Several diagnostic methods are used in the diagnosis and differential diagnosis of osteoporosis, which can be divided into the following:

- **1)** **Basic examinations**
  **- X-ray examination**
  - X-ray of the Th and LS spine in a lateral projection to evaluate deformities in patients with an increased risk of vertebral fractures,
  - X-ray of the skeleton (spine, pelvis, skull, upper limbs, and lower limbs) in the diagnosis of fractures and in the differential diagnosis of other skeletal diseases.
  - **2)** **Specialized**
  - QCT
  - Computed tomography (CT) and magnetic resonance (MR) examination.
  - Scintigraphy of the skeleton.
  - Bone biopsy.

The diagnosis of osteoporosis can be established if a low-traumatic fracture of the vertebra or proximal femur is present (independent of BMD). The diagnosis of osteoporosis can be established if the measured T-score value is from − 1.0 to − 2.5 SD and at the same time:

- Low-traumatic fracture of the proximal humerus, pelvis, or distal forearm.

- Multiple low-traumatic fractures in other places (except the face, feet, and hands).

Low-traumatic (osteoporotic) fractures can be considered those that occurred *without known trauma or after an inadequately small trauma* (this can be defined as a fall from a height no higher than a fall from a standing position). When evaluating a fracture, it is therefore necessary to take the mechanism of the injury into account [7].

### X-ray examination in the diagnosis of osteoporosis

The classification of fractures in the spine is based on the assessment of changes in the height of the Th and LS vertebral bodies in the lateral projection using the semi-quantitative method in accordance with Genant [9]. The method will also enable the classification of vertebral fractures. A reduction in the height of the vertebral body by more than 20% (anterior, posterior edge, or center) is considered a grade 1 fracture or mild fracture. A height reduction of more than 25% and less than 40% indicates moderate fracture (grade 2 fracture). A reduction of more than 40% indicates a grade 3 fracture, or a severe fracture.

### Lateral imaging scan of the spine using the DXA method

To assess the deformities of the vertebrae, it is also possible to use an imaging examination of the spine in a lateral projection using the DXA method [10]. It is a quick examination with minimal radiation exposure; it enables—as does X-ray—assessment of vertebral deformities based on Genant’s classification***.***

### CT or MR examination in the diagnosis of osteoporosis

A CT or an MR examination of the spine is indicated if it is not possible to clearly detect a decrease in the height of the vertebral body from a classic X-ray examination or lateral DXA scan (e.g., scoliosis, condition after spine surgery). They are also recommended when clinical symptoms and other tests suggest that the decrease in vertebral body height may be due to a cause other than osteoporotic vertebral deformity.

## Fracture risk calculator: FRAX

FRAX is a computer algorithm that calculates the 10-year probability of any major fracture (hip, spine, humerus, or wrist) and the 10-year probability of a proximal femur fracture. Fracture risk is calculated based on age, BMI, and risk factors that include past osteoporotic fracture, family history of proximal femur fracture, smoking, long-term glucocorticoid use, rheumatoid arthritis, other causes of secondary osteoporosis, and alcohol consumption. The BMD value of the proximal femur can be added to increase fracture risk prediction.

When calculating risk using FRAX, we can use three methods:

- FRAX without BMD
- FRAX using BMD
- FRAX adjusted to TBS

Patients with a FRAX ≥ 20% for any major osteoporotic fracture and/or a FRAX ≥ 3% for a proximal femur fracture are considered high risk [11].

The FRAX specific to the Slovak population is on the website: https://www.sheffield.ac.uk/FRAX/tool.aspx?country=44.

### Differential diagnosis

The cause of low bone density needs to be evaluated in every patient. If—based on the history, objective findings, and the results of basic laboratory and imaging examinations—it can be assumed that low bone density, in addition to age, or other risk factors also play a role in estrogen deficiency, it is necessary to rule out secondary osteoporosis and other metabolic diseases of the skeleton, which may be accompanied by low bone density. These are mainly patients under the age of 50. In older patients, it is necessary to think about secondary osteoporosis, especially if the bone density is significantly lower than the expected value for the given age, that is, if the Z-score value is lower than − 2.0 SD and in patients with a rapid decrease in BMD.

Differential diagnosis includes the exclusion of all diseases that lead to secondary osteoporosis or low bone density (Table 2) with the use of all necessary laboratory and imaging methods in accordance with the assumed diagnosis.

**Table 2:** Specialized laboratory examinations in the differential diagnosis of osteoporosis

Erythrocyte sedimentation rate (FW)
Cortisol in urine in 24 h
Follicle-stimulating hormone (FSH), luteinizing hormone (LH)
Prolactin
Thyroid stimulating hormone (TSH)
Parathyroid hormone (PTH)
Serum magnesium
1,25-Dihydroxy-vitamin D
Rheumatoid factor, ASLO
Ferritin, serum iron, transferrin, iron binding capacity
Homocysteine
Screening for celiac disease (antibodies against tissue transglutaminase (anti-TTG), antibodies against endomysium (EMA), and antibodies against deaminated gliadin peptides (DGP))
COL1 A gene (for diagnosis of osteogenesis imperfecta)
Tryptase and histamine levels

A bone biopsy is indicated if other methods cannot clarify the etiology of low density, repeated fractures, or it is necessary to exclude other metabolic or tumor involvement of the bones.

### Prevention

As part of the prevention of osteoporosis and osteoporotic fractures, it is necessary to exclude known risk factors (Table 1), to reduce the risk of falls. Patients should be recommended a physically active lifestyle, maintaining a BMI above 19 kg/m^2^ and weight-bearing exercises adapted to the needs and abilities of the patient. An adequate supply of protein in the diet is recommended. It is necessary to ensure an adequate daily intake of calcium. The recommended dose of calcium in accordance with age, gender, and hormonal status is in Suppl. Table 8. If it is not possible to ensure the recommended amount of calcium in the diet, supplementation in the form of calcium preparations is suitable. Vitamin D supplementation is recommended for patients with a proven vitamin D deficiency as well as for people with a presumed deficiency (elderly patients, especially with insufficient exposure to sunlight and in the winter months). The goal of supplementation is to ensure an optimal level of 25-OH-D3 vitamin (≥ 30 ng/mL or ≥ 75 nmol/L). The recommended daily dose of cholecalciferol is 800 to 1000 IU (see also Suppl. Table 9). In patients with chronic kidney disease, the administration of vitamin D analogues is an alternative [12] (Table 3).

**Table 3:** Indications for a lateral X-ray image and for a lateral DXA scan

• Clinical suspicion of a fracture
• Suspicion of a fracture from a DXA scan in the AP projection when measuring bone density
• Increased fracture risk defined on the basis of the FRAX calculator (with/without BMD)
• Decrease in height by more than 4 cm compared with the height in youth
• Vertebral fracture reported by the patient, not documented until then
• Glucocorticoid therapy ≥ 5 mg of prednisone or has an equivalent dose of another glucocorticoid daily for ≥ 3 months, or a cumulative dose of 2.7 g

Secondary prevention of fractures is focused on patient care after osteoporotic fractures. Patients after treatment of an osteoporotic fracture of the forearm (after the age of 50) as well as patients with a diagnosed osteoporotic fracture of the vertebra should be referred by the attending physician (orthopedic, traumatologist, surgeon, or emergency medicine physician) for examination in the outpatient clinic of a specialist focused on diagnosis, differential diagnosis, and osteoporosis treatment [13, 14]. After treatment of osteoporotic fracture of the proximal femur, it is necessary to provide calcium and vitamin D supplementation during hospitalization (unless there are contraindications to their administration); and upon discharge, the patient is sent for examination in the outpatient clinic of a specialist focused on diagnosis, differential diagnosis, and treatment of osteoporosis.

### Treatment

The goal of osteoporosis treatment is to reduce the risk of fracture. Adequate supplementation of calcium and vitamin D is the basis of prevention and treatment. Ensuring the daily requirement of calcium and the ideal level of vitamin D is also necessary when taking antiresorptive and/or osteoanabolic treatment. The treatment is long-term, usually lifelong, considering the contraindications of this treatment.

Drugs that have been proven to reduce fracture risk include antiresorptives (bisphosphonates such as alendronate, ibandronate, risedronate, and zoledronic acid, as well as denosumab and raloxifene), osteoanabolics (such as teriparatide), and mixed-action therapies (such as romosozumab) (see Table 4) [15].

**Table 4:** Efficacy of antiporotic medications

Antiporotic treatment	Effect on vertebral fractures		Effect on non-vertebral fractures		Osteoporosis in men	GIOP
	Osteoporosis	Prevention of further fractures	Osteoporosis	Prevention of further fractures		
Alendronate	+	+	*	_+_a)	+	+
Risedronate	+	+	*	_+_a)	+	+
Ibandronate	NA	+	NA	_+_ b)	NA	NA
Zoledronic acid	+	+	NA	_+_c)	+	+
Denosumab	+	_+_ c)	_+_a)	_+_c)	_+_d)	+
HRT	+	+	+	_+_a)	NA	NA
Raloxifene	+	+	NA	NA	NA	NA
Romosozumab	+	+	+	+	NA	NA
Teriparatide	NA	+	NA	+	+	+

Explanations: +, proven clinical effect; *NA*, no proven EBM effect; *, presumed efficacy but not clinically proven; *GIOP*, glucocorticoid-induced osteoporosis; *HRT*, hormone replacement therapy (estrogens).

^a)^Including hip fracture.

^b)^Only in some patients in accordance with post hoc analyses.

^c)^Mixed group of patients with or without prevalent vertebral fracture.

^d)^Indicated in men with prostate cancer after hormonal ablation.

## Bisphosphonates

These are synthetic analogues of pyrophosphates, which are resistant to the action of endogenous pyrophosphatases. They have a high affinity for the bone mineral of the bone surface [16]. Their action ultimately leads to dysfunction or even apoptosis of osteoclasts. Bone resorption decreases, and depending on the type of bisphosphonate, impaired bone remodeling may occur. The ability to inhibit bone resorption is based on interference with the enzyme activity of osteoclasts; they inhibit the enzyme *farnesyl diphosphate synthase* (synonym *farnesyl pyrophosphate synthase*) in the 3-hydroxy- 3-methylglutaryl coenzyme A reductase pathway, important in the process of GTPase activation. GTPases are signaling proteins that when activated positively regulate structural properties and processes important for the function of osteoclasts: morphology, cytoskeleton formation, vesicular functions, and membrane folding. The potential for inhibition of farnesyl pyrophosphate synthase decreases in the order “zoledronate > risedronate ˃ ibandronate ˃ alendronate.”

Alendronate, risedronate, ibandronate, and zoledronic acid are indicated in the treatment of postmenopausal osteoporosis.

Treatment with bisphosphonates is indicated in these patients:

- DXA-verified osteoporosis based on World Health Organization (WHO) criteria.
- Low-traumatic fracture of the vertebra or proximal femur.
- Postmenopausal women who have a T-score of − 1.0 to − 2.5 SD and a history of fracture of the proximal humerus, pelvis, or distal forearm or a history of multiple fractures at other sites (except the face, feet, and hands).
- Treated with glucocorticoids for more than 3 months and in a dose of more than 5 mg of prednisone (or its equivalent), or a cumulative dose of 2.7 g of prednisone per year if the T-score value in the spine or femur is less than − 2.0 SD.
- Both women and men with BMD in the osteopenia range and at the same time a high fracture risk defined based on the FRAX calculator as a 10-year risk of major osteoporotic fracture ≥ 20% or risk of femur fracture ≥ 3%

Alendronate, risedronate, and zoledronic acid are indicated for the treatment of glucocorticoid-induced osteoporosis (GIOP).

Alendronate, risedronate, and zoledronic acid are indicated for the treatment of osteoporosis in men. The use of zoledronic acid in oncological indications is beyond the scope of this standard.

Before starting treatment with bisphosphonates, it is necessary to inform the patient about the potential risks of treatment:

- Osteonecrosis of the jaw during treatment and the need to observe proper oral hygiene undergo regular dental examinations or the need to inform the dentist about ongoing treatment before planned dental surgery
- Atypical fractures of the femur, especially during their long-term use

It is also necessary to consider all gastrointestinal, renal, and other contraindications to the use of bisphosphonates in accordance with the current summary of product characteristic (SPC) of individual bisphosphonates.

Oral bisphosphonate treatment should generally be continued for 5 years. For intravenous zoledronic acid, a minimum of 3 years of treatment is recommended. After 3 to 5 years of bisphosphonate therapy, the patient’s fracture risk should be reassessed. If the risk is low, treatment may be discontinued, with regular monitoring (via DXA and bone turnover markers) to track the patient’s status. For patients with high or very high fracture risk, long-term bisphosphonate therapy is recommended (see Table 5). The upper limit for the duration of bisphosphonate treatment in high-risk patients is not well-defined, whereas 5 years is suggested; briefer treatment durations may also be appropriate depending on individual risk factors.

**Table 5:** Evaluation of the patient’s fracture risk profile

Very high risk	High risk	Low risk
• Fracture within the last 12 months • Multiple fractures • Fractures during osteoporosis treatment • Fractures during treatment that negatively affects the bone • Very low T-score − 3.0 SD • FRAX ≥ 30% for a large osteoporotic fracture, ≥ 4.5% for hip fracture	• Age ≥ 65 years • Overcome fracture in ≥ 12 months • T-score ≤ − 2.5 SD • T-score − 1.0 to − 2.5 SD and FRAX ≥ 20% for large osteoporotic fracture or ≥ 3% for hip fracture	• Age after menopause • Without a previous fracture • T-score ≥ − 1.0 SD and FRAX < 20% for a large osteoporotic fracture or < 3% for a hip fracture

A patient is considered at risk if at least one of the listed risk factors is present.

The length of treatment interruption is individual and depends on the type of bisphosphonate and on the development of bone density and changes in markers of bone turnover. Restarting treatment or starting treatment with a different mechanism of action is appropriate when:

- BMD decreases by more than the LSC
- Significant increase in bone turnover markers occurs
- New low-traumatic fracture occurs

Dosage, method of administration of bisphosphonates, and their contraindications are governed by valid SPCs for individual drugs.

## Denosumab

Denosumab is a recombinant, fully human monoclonal antibody against RANKL (receptor activator for nuclear factor kappa-b ligand): a key mediator of osteoclast differentiation, function, and survival. There is evidence-based medicine (EBM)-proven risk reduction of vertebral, non-vertebral, and femoral neck fractures [17]. Treatment with denosumab is indicated in postmenopausal women and in men with:

- DXA-verified osteoporosis based on WHO criteria
- Low-traumatic fracture of the vertebra or proximal femur
- Postmenopausal women who have a T-score from − 1.0 to − 2.5 SD and a history of fracture of the proximal humerus, pelvis, or distal forearm or a history of multiple fractures at other sites (except the face, feet, and hands)
- Both women and men with bone density in the osteopenia range and at the same time a high risk of fracture defined based on FRAX calculator as a 10-year risk of a major osteoporotic fracture of more than 20% or a risk of femur fracture of more than 3%
- Women and in men treated with glucocorticoids for more than 3 months and in a dose of more than 5 mg of prednisone (or its equivalent) or a cumulative dose of 2.7 g of prednisone per year, if the value of the T-score in ​​the spine or femur is less than − 2.0 SD
- Men with non-metastatic prostate cancer who are treated with ADT and have a BMD T-score at the lumbar spine, total hip, or femoral neck < − 1.0 SD or with a history of osteoporotic fracture

Before starting treatment with denosumab, patient should be informed about these risks:

- Skipping the application or interrupting the treatment (risk of rebound phenomenon with multiple vertebral fractures).
- Osteonecrosis of the jaw and its prevention in the sense of observing the previous correct oral hygiene, absolving regular dental check-ups, or the need to inform the dentist about the current treatment with denosumab before a planned dental surgery.
- Atypical femur fractures.

Treatment with denosumab is long-term. The efficacy and safety of the treatment has been demonstrated for at least 10 years of continuous administration. Patients with a high risk of developing osteoporotic fractures (Table 5) can continue treatment with denosumab for a long time. If it is not possible to administer additional denosumab, another anti-osteoporotic treatment should be continued. In patients with moderate and low risk of developing osteoporotic fractures, discontinuation of treatment may be considered after 5 to 10 years of continuous treatment with denosumab.

**Fig. 1:**
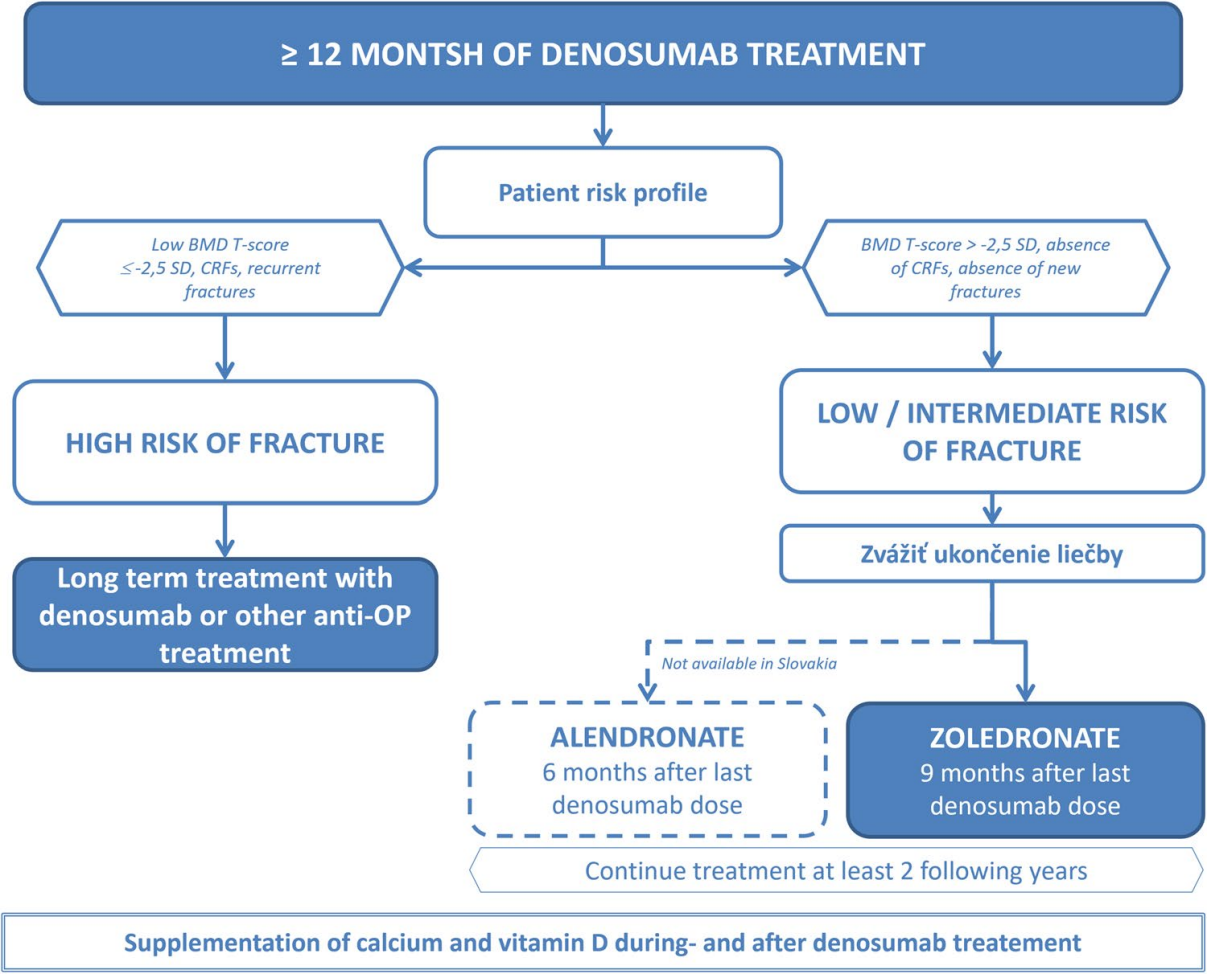
Denosumab treatment algorithm

If patients have been treated with denosumab for at least 12 months, it is necessary to administer antiresorptive treatment with bisphosphonates to eliminate the possible rebound phenomenon [18] (Fig. 1). The oral bisphosphonate alendronate should be started 6 months after the last dose of denosumab. Parenteral administration of zoledronic acid is appropriate 9 months after the last dose of denosumab. Treatment with bisphosphonates after stopping denosumab should last 2 years with regular controls of bone density and laboratory markers of bone metabolism.

In patients with multiple vertebral fractures due to the rebound phenomenon, based on the data published so far, vertebroplasty or kyphoplasty is not appropriate.

## Selective estrogen receptor modulators

Selective estrogen receptor modulators (SERMs) are substances that act as strong estrogen agonists on the bone and cardiovascular apparatus and at the same time have an antagonistic effect on breast tissue. They have an EBM-documented effect on the reduction of vertebral fractures [19]. They are indicated for the treatment of osteoporosis in postmenopausal women with:

- BMD-verified osteoporosis based on WHO criteria.
- Low-traumatic fracture of the vertebra or proximal femur.
- Patients with bone density in the osteopenia range and at the same time a high risk of fracture defined based on FRAX calculator as a 10-year risk of a large osteoporotic fracture of more than 20% or a risk of femur fracture of more than 3%

The benefit of the treatment is also in patients with osteoporosis and a high risk of breast cancer. Treatment is not suitable for patients with climacteric syndrome and at risk of thromboembolism.

Dosage, method of administration, and contraindications are determined by the valid SPC. The duration of treatment is not determined; after 5 years of treatment, it is appropriate to re-evaluate the patient’s risk profile. At high risk, continuation of treatment or change of treatment with a different mechanism of action is possible.

## Hormone replacement therapy

### Estrogen therapy in women

Hormone replacement therapy (HRT) has an EBM-documented effect on the reduction of vertebral fractures as well as proximal femur fractures [20]. Due to the increased risk of invasive breast cancer, thromboembolic events, and cardiovascular and cerebrovascular complications, HRT is not indicated for the treatment of osteoporosis [21]. HRT is indicated for the treatment of climacteric syndrome.

### Androgen therapy in men

Androgen treatment has a beneficial effect on bone tissue, but its effect on fracture reduction has not been sufficiently demonstrated. Specific anti-osteoporotic treatment is also indicated for the treatment of osteoporosis in men with hypogonadism.

## Teriparatide (1.34 PTH)

Continuous hypersecretion of parathyroid hormone has a catabolic effect on the skeleton, leading to bone destruction and the development of osteoporosis, but as early as 1929, an increase in the number of bone beams was achieved by intermittent application of an extract from the parathyroid glands. The basis of the diametrically different effect of continuous hypersecretion of parathyroid hormone and intermittent daily application on bone lies in the different regulation of gene expression. A single application leads to a rapid but transient increase in RANKL expression and a decrease in osteoprotegerin expression. This effect lasts for 24 h. The osteoanabolic effect consists of an increase in bone mass, an increase in the number of osteoblasts, and an increase in bone strength. Teriparatide reduces the risk of vertebral and non-vertebral fractures [22]. Treatment is reserved for severe osteoporosis and is governed by current indication criteria.

The indication for treatment with teriparatide is postmenopausal osteoporosis in women and osteoporosis in men with a DXA determined T-score < − 2.5 SD in the femoral neck or total hip area or in the L-spine area + one of the following criteria:

- Verified vertebral fracture after inadequate trauma,
- Long-term treatment with glucocorticoids (more than 3 months in a dose of more than 5 mg of prednisone or in an equivalent dose of another glucocorticoid or a cumulative dose of 2.7 g of prednisone per year).
- If after 2 years of antiresorptive treatment there was a decrease in bone density by more than the LSC value in the given area or they suffered an osteoporotic fracture.

The duration of treatment is 18 to 24 months. After the end of the treatment, the use of antiresorptive therapy is recommended.

## Romosozumab

Romosozumab is a humanized monoclonal antibody (IgG2) that binds and inhibits sclerostin, thereby increasing bone formation by activating bone lining cells, increasing bone production by osteoblasts and recruitment of osteoprogenitor cells. Romosozumab also affects changes in the expression of osteoclast mediators, thereby reducing bone resorption. Together, this dual effect of increasing bone formation and decreasing bone resorption leads to a rapid increase in trabecular and cortical bone mass, improving bone structure and strength.

New vertebral fractures and clinical fractures were significantly reduced in women treated with romosozumab when compared with placebo at 12 months. After switching both arms to denosumab, at 24 months, vertebral fracture rates were significantly lower in women treated with romosozumab during the first 12 months [23]. Significantly greater risk reduction in new vertebral and clinical fractures was seen for romosozumab vs. alendronate at 12 months [24].

The indication for romosozumab treatment is postmenopausal osteoporosis in women with a DXA-determined T-score < − 2.5 SD in the femoral neck or total hip area or in the L-spine area + one of the following criteria:

- Verified vertebral fracture after inadequate trauma.
- If after 2 years of antiresorptive treatment there was a decrease in bone density by more than the LSC value in the given area or they suffered an osteoporotic fracture.

Covered treatment can be indicated and the drug prescribed only in selected centers.

Covered treatment is time-limited to one 12-month cycle.

### Monitoring of osteoporosis treatment using bone markers

A significant association was demonstrated between the decrease in bone turnover markers and the reduction in fracture risk. Significant change in bone turnover markers after 3–6 months of treatment, e.g., a decrease in CTx in antiresorptive therapy and an increase in P1 NP or osteocalcin in osteoanabolic therapy by more than the LSC value [25–27].

If no significant change in bone turnover is observed, the physician should reassess:

- Adherence to the regimen and the correct use of treatment (compliance and adherence),
- Insufficient resorption.
- Exclusion of secondary osteoporosis.

### Monitoring of osteoporosis treatment using densitometry

An increase in bone density is significantly correlated with a decrease in fracture risk. The measurement of bone density is, therefore, important in evaluating the rate of bone loss and in evaluating the effectiveness of treatment. Control measurement of bone density is important if a change in density is expected higher than the value of the smallest significant change determined at the given workplace. After the initiation of antiresorptive or osteoanabolic therapy, a significant increase in bone density is expected at the earliest after 1 year of treatment and depends on the type of treatment.

After proving the effectiveness of the applied treatment, the measurement interval can be extended, provided that the treatment is continued (adherence to the treatment) and the patient’s risk profile does not change significantly.

If the expected increase in BMD does not occur, it is appropriate to exclude the causes of insufficient increase in BMD.

However, the treatment can be considered effective in terms of reducing the risk of fractures if there is no decrease in BMD by more than the LSC value determined at the given workplace.

### Osteoporosis treatment failure

Treatment failure is considered if one of the following is present:

- For antiresorptive drugs, CTx and P1 NP decrease by less than the LSC (if baseline values are not known, positive response = less than mean value of young healthy adults).
- For teriparatide, the increase of bone markers is lower than the LSC.
- A decrease in BMD more than the LSC value determined at the workplace.
- The occurrence of a second osteoporotic fracture during treatment is a reason to reassess the effectiveness of treatment (the duration of treatment, the risk profile of the patient, and the mechanism leading to the fracture must also be considered) [28].
- Specific management of patients with low bone density in oncologic diseases and their treatment is determined by specific recommendations.

### Sequential treatment in osteoporosis

Sequential treatment in postmenopausal osteoporosis represents an evolving therapeutic approach aimed at maximizing bone health and reducing fracture risk in this vulnerable population. This strategy typically begins with anabolic agents, such as teriparatide or abaloparatide, which stimulate bone formation and increase in BMD. Following a defined anabolic treatment period, patients transition to antiresorptive agents—including bisphosphonates (e.g., alendronate) or denosumab—to maintain and further enhance the bone gains achieved during the anabolic phase.

Clinical trials and meta-analyses have consistently demonstrated the superiority of sequential treatment compared with monotherapy [29]. For example, initiating therapy with an anabolic agent and subsequently switching to antiresorptive medication results in greater improvements in BMD at critical skeletal sites—including the lumbar spine and femoral neck—compared with using antiresorptive therapy alone. Moreover, this approach has been associated with a sustained reduction in vertebral, non-vertebral, and hip fractures; which are common and debilitating consequences of osteoporosis. Romosozumab has emerged as a pivotal anabolic agent in the sequential treatment of postmenopausal osteoporosis. Its dual action of increasing bone formation and decreasing bone resorption positions it as an effective option for initiating therapy in high-risk patients, particularly those with severe osteoporosis or multiple fractures. Clinical trials have demonstrated that transitioning from romosozumab to antiresorptive agents, such as alendronate or denosumab, results in substantial and sustained improvements in bone mineral density and fracture risk reduction [30].

Recent evidence highlights the importance of timing and patient selection in sequential therapy. In cases of severe osteoporosis or patients with multiple fractures, early initiation with anabolic therapy can provide a robust foundation for long-term bone health. Conversely, initiating with antiresorptive agents and subsequently transitioning to anabolic agents may result in less pronounced BMD gains, underscoring the need for a tailored approach based on individual fracture risk and baseline bone density [31].

The long-term success of sequential treatment also hinges on adherence to therapy and monitoring. Emerging research into novel therapeutic combinations and more-targeted interventions continues to refine these strategies, promising better outcomes for postmenopausal women at high risk for fractures.

### *Opinion of experts (assessment activity, revision activity, caregivers, *etc*.)*

Medical examiners of the Social Insurance Company assess the health status of patients for the purposes of disability and determine the percentage rate of decline in the ability to perform gainful activity in accordance with the type of disability of organs and systems, to Law 461/2003 Coll. on Social Insurance, as amended and valid from August 1, 2023. Osteoporosis is included in Chapter 15 “Diseases of the musculoskeletal system,” Section B “Osteopathy and chondropathy,” and Item 1 “Osteoporosis (regardless of etiology).”

Since the philosophy of this law is based on the severity of the functional disability of the organ with an adverse effect on the overall performance of the organism, osteoporosis is divided in this law into:

- Mild form: osteoporosis defined based on T-score below − 2.5, without low-traumatic fractures, but increasing the risk of fractures and affecting the body’s performance. In accordance with the law, this form stipulates a percentage decrease in the ability to perform gainful activity in the range of 10 to 15%.
- Moderately severe form: the presence of one or more low-traumatic fractures due to osteoporosis (forearm, humerus, spine, femur, or other fractures resulting from an inadequately small trauma defined as a fall from a height no greater than a fall from a standing position or without it) regardless of value of bone density but with subsequent mild or moderate limitation of mobility, weakness of the muscle corset, and a decrease in performance, affecting the statics of the spine or persistent pain syndrome due to an overcome fracture. In accordance with the law, this form conditions the percentage rate of decline in the ability to perform gainful activity in the range of 35 to 45% (considering the type of work performed).
- Severe form of osteoporosis: occurrence of low-traumatic fractures (vertebrae, pelvis, proximal femur, and others) caused after an inadequately small trauma defined as a fall from a height no greater than a fall from a standing position or even without it) regardless of the value of ankle density with subsequent serious functional impairment abilities (deformation of the spine with subsequent severe limitation of mobility of the spine or significant affection of mobility after a fracture of the femur or persistent significant pain after an overcome fracture and/or also manifestations of nerve and muscle irritation due to weakness of the muscle corset), where the percentage rate of decrease in the ability to perform gainful activity is determined by a range of 60 to 75% (the type of work performed is taken into account).

Secondary osteoporosis is assessed for disability purposes in relation to the underlying disease.

The decline in the ability to perform a gainful activity is assessed by medical examiners on the basis of the submitted medical reports and data from the health documentation of the medical facility with the determination of the diagnostic conclusion, the development of the disease, the success of the treatment, complex functional examinations, and their conclusions, yet taking into account the remaining ability to perform a gainful activity and the impact of the disease on the overall state of the organism.

## Supplementary Information

Below is the link to the electronic supplementary material.

Supplementary file1 (PNG 204 KB)

Supplementary file2 (DOCX 17 KB)

## Abbreviations

**ADT**: Androgen deprivation therapy
**ALP**: Alkaline phosphatase
**ALT**: Alanine aminotransferase
**anti-TTG**: Antibodies against tissue transglutaminase
**AP**: Antero-posterior
**ASLO**: Antistreptolysin O
**AST**: Aspartate aminotransferase
**BMD**: Bone mineral density
**BMI**: Body mass index
**CKD–MBD**: Chronic kidney disease–mineral and bone disorder
**CRP**: C-reactive protein
**CT**: Computed tomography
**CTX**: Carboxy-terminal collagen crosslinks
**DGP**: Antibodies against deamidated gliadin peptides
**DM**: Diabetes mellitus
**DXA**: Dual-energy X*-*ray absorptiometry
**EBM**: Evidence-based medicine
**ELFO**: Protein electrophoresis
**EMA**: Endomysia antibodies
**ESCEO**: European Society for Clinical and Economic Aspects of Osteoporosis and Osteoarthritis
**FRAX**: Fracture Risk Assessment Tool
**FSH**: Follicle-stimulating hormone
**FW**: Erythrocyte sedimentation rate
**GIOP**: Glucocorticoid-induced osteoporosis
**GMT**: Gamma-glutamyl transferase
**HRT**: Hormone replacement therapy
**IOF**: International Osteoporosis Foundation
**ISCD**: International Society for Clinical Densitometry
**L1–L4**: Lumbar vertebra 1 to 4
**LH**: Luteinizing hormone
**LS**: Lumbo-sacral
**LSC**: Least significant change
**MR**: Magnetic resonance
**PTH**: Parathyroid hormone
**QC**: Quality control
**QCT**: Quantitative computed tomography
**RTG**: Roentgen (X-ray in English literature)
**SD**: Standard deviation
**SERM**: Selective modulator of estrogen receptors
**SPC**: Summary of product characteristic
**TBS**: Trabecular bone score
**TSH**: Thyroid-stimulating hormone
**USPTF**: United States Preventive Services Task Force
**WHO**: World Health Organization
