# Supplementary figures and images for: National guidelines for diagnosis and treatment of osteoporosis in Slovakia
Source: Arch Osteoporos. 2025 May 4;20(1):56. doi: 10.1007/s11657-025-01538-z (PMC12050228; doi:10.1007/s11657-025-01538-z)

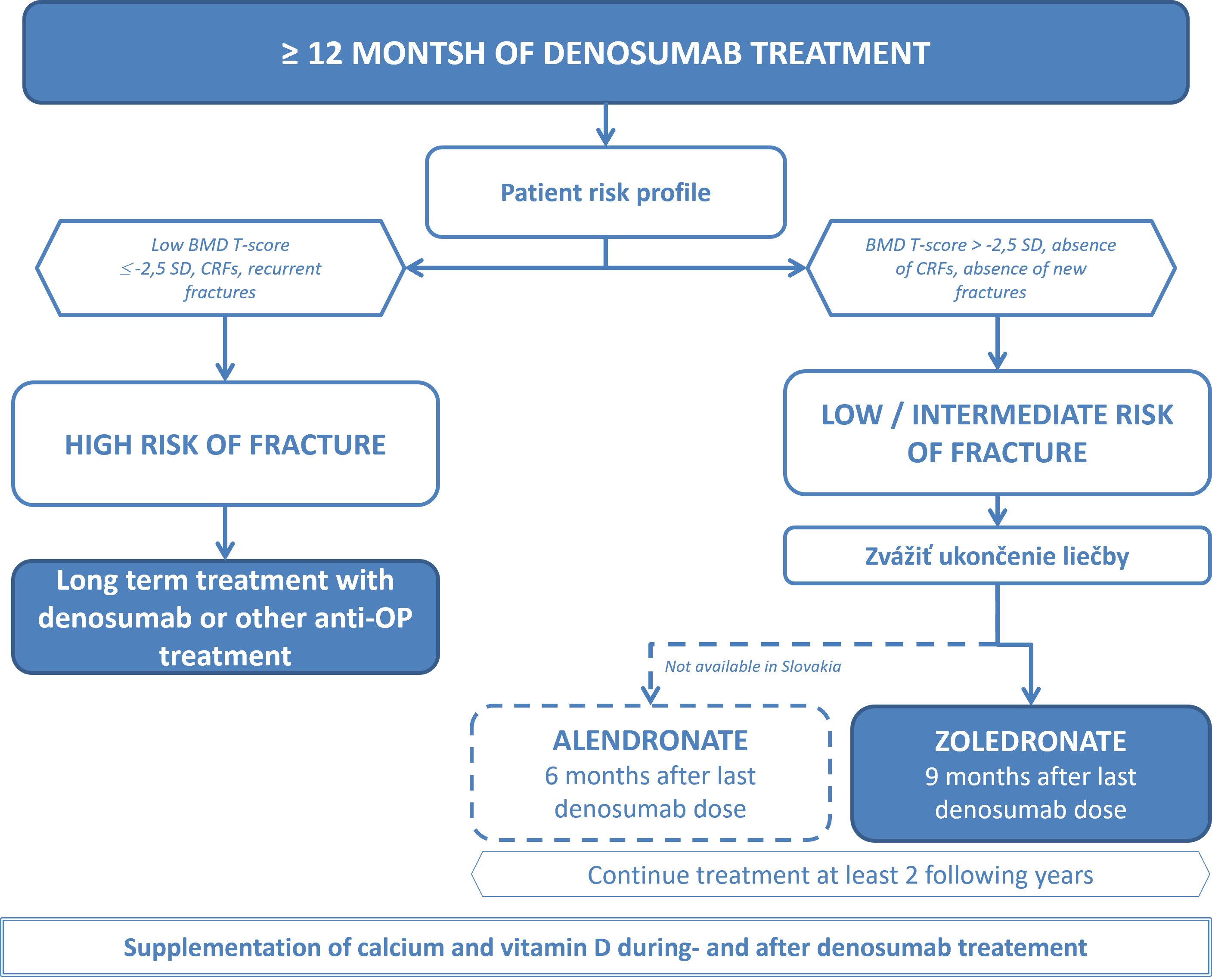

Supplement: Supplementary file 1 — Supplementary file1 (PNG 204 KB) [file 11657_2025_1538_MOESM1_ESM.png]
