# Supplementary material for: National guidelines for diagnosis and treatment of osteoporosis in Slovakia
Source: Arch Osteoporos. 2025 May 4;20(1):56. doi: 10.1007/s11657-025-01538-z (PMC12050228; doi:10.1007/s11657-025-01538-z)
Supplement: Supplementary file 2 — Supplementary file2 (DOCX 17 KB) [file 11657_2025_1538_MOESM2_ESM.docx]

**Supplementary information**

Suppl. Table 3. Indications for DXA examination in Slovakia.

Suppl. Table 4. WHO diagnostic criteria for postmenopausal women and men over 50 years of age.

Suppl. Table 5. Requirements for the densitometric evaluation report.

Suppl. Table 6. Requirements for the report from the evaluation of the follow-up densitometric examination.

Suppl. Table 8. Recommended optimal daily intake of calcium.

Suppl. Table 9. IOF, ESCEO, and USPTF recommendations for calcium and vitamin D supplementation.
